# Multimodal modulation of metal-based nanomaterials in osteoarthritis immunotherapy: current landscape and future paradigms

**DOI:** 10.3389/fimmu.2026.1827369

**Published:** 2026-07-03

**Authors:** Jiayi Chen, Jun Ma, Zhuoming Xu, Huanhuan Luo, Haodong Hu, Chenhong Qian, Kang Ji

**Affiliations:** Jiaxing Key Laboratory of Basic Research and Clinical Translation on Orthopedic Biomaterials, Department of Orthopedics, The Second Affiliated Hospital of Jiaxing University, Jiaxing, China

**Keywords:** immunometabolic regulation, immunotherapy, metal-based nanomaterials, nanoparticle targeting, osteoarthritis

## Abstract

Osteoarthritis (OA) is a degenerative joint disease characterized by immune-metabolic dysregulation, oxidative stress, and chronic inflammation. Current therapies offer symptomatic relief but do not halt disease progression. Metal-based nanomaterials (MNMs) have recently emerged as innovative immunomodulatory agents for OA treatment. This review summarizes key immune-pathological mechanisms in OA, such as macrophage polarization imbalance, NLRP3 inflammasome activation, and ferroptosis, and examines how MNMs mitigate these processes via ROS scavenging, macrophage reprogramming, and targeted inhibition of inflammatory signaling. We also highlight advanced material design strategies such as surface functionalization and stimulus-responsive drug release for improved targeting and efficacy. Despite promising preclinical results, challenges related to long-term biosafety and clinical translation remain. Future efforts should focus on rational nanomaterial design, mechanistic studies using advanced omics technologies, and the development of more physiologically relevant disease models to facilitate clinical application.

## 1 Introduction

Osteoarthritis (OA), the most prevalent degenerative joint disease, is characterized by core pathological features including cartilage degradation, synovitis, and osteophyte formation, posing a significant challenge to global public health (1–3). According to the Global Burden of Disease Study, the prevalence of OA has risen substantially over the past three decades, with this trend being particularly pronounced in the Asia-Pacific region due to its accelerated population aging (4). It is noteworthy that OA is not only a leading cause of chronic pain and motor dysfunction in middle-aged and elderly populations, but also imposes a substantial economic burden on individuals, families, and society at large due to its high disability rate (5).

Current management strategies for OA primarily focus on pain relief and improvement of joint function. The established stepwise clinical approach, guided by disease severity, encompasses basic interventions, pharmacotherapy, reparative procedures, and joint reconstruction (6–8). Basic treatment is indicated for patients with mild OA symptoms, encompassing health education, weight management, appropriate exercise, and physical therapy (9, 10). In early-stage OA, pharmacological treatment typically relies on nonsteroidal anti-inflammatory drugs (NSAIDs) as first-line agents (11). However, prolonged NSAID use is associated with risks of gastrointestinal mucosal injury, renal impairment, and cardiovascular events, while its analgesic efficacy tends to decline as the disease advances (12). For moderate to severe cases, intra-articular injections of glucocorticoids or hyaluronic acid derivatives offer short-term symptom relief but do not arrest cartilage degeneration; repeated administration may even accelerate pathological subchondral bone remodeling (13, 14). More concerning is the overprescription of opioids, which has precipitated a significant dependency crisis. Data indicate that rates of persistent postoperative opioid use are markedly higher in OA patients than in those with other chronic pain conditions (15). When symptoms remain refractory to basic and pharmacological measures, reparative or reconstructive interventions are considered. These aim to restore joint function and reduce pain through cartilage repair or replacement, removal of pathological intra-articular tissues, and improvement of mechanical alignment and stability. Common reparative approaches include arthroscopic debridement, autologous platelet-rich plasma (PRP) injections, stem cell therapy, and cartilage transplantation (16–20). For patients with severe osteoarthritis or those with end-stage disease refractory to other treatments, total joint arthroplasty serves as the primary intervention; Although it effectively restores joint function (2, 21), long-term follow-up data reveal a substantial rate of prosthetic loosening within 10 years postoperatively. Moreover, revision surgery carries a significantly higher risk of complications compared to primary procedure (22). High treatment costs further limit its widespread applicability (23).

Notably, current therapeutic regimens predominantly address symptom alleviation rather than modifying the underlying disease pathology (24). The pathogenesis of osteoarthritis (OA) is complex and multifactorial, with immune-metabolic dysregulation being increasingly recognized as a central pathomechanism (25). While OA has traditionally been classified as a degenerative and biomechanical disease, an expanding body of evidence demonstrates that aberrant immune activation, particularly involving innate immune sensors and effector cells, actively propagates cartilage degradation and synovial inflammation. This immunopathologic component transforms OA from a purely mechanical wear-and-tear process into a complex disease with autoinflammatory features, thereby justifying immunomodulatory therapeutic strategies. Accumulating evidence indicates that OA is not a passive consequence of mechanical wear or aging, but an active biological process driven by multifactorial interactions, where dynamic shifts in the local immune microenvironment critically govern disease initiation and progression (26, 27). Specifically, excessively activated M1-polarized synovial macrophages drive the expression of cartilage-degrading enzymes (e.g., MMP-13, ADAMTS-5) via the release of pro-inflammatory cytokines such as IL-1β and TNF-α, while an imbalance in the Treg/Th17 cell ratio further perpetuates chronic inflammation (28–31). Concurrently, the accumulation of reactive oxygen species (ROS) within the joint induces mitochondrial dysfunction, promoting chondrocyte ferroptosis and establishing a self-sustaining cycle of inflammation and oxidative stress (32–34). Existing pharmacologic agents lack the targeting specificity required to disrupt this pathological circuit, creating a significant therapeutic impasse. This limitation underscores the urgent need for novel interventions. Consequently, disease-modifying osteoarthritis therapy (DMOAT), predicated on multi-targeted modulation of the immune microenvironment, has emerged as a pivotal research frontier aimed at decelerating disease progression and potentially reversing structural damage (35).

In light of the persistent challenge in balancing efficacy and safety with conventional OA treatments, MNMs are emerging as innovative candidates for disease-modifying osteoarthritis therapy (DMOAT) by capitalizing on their intrinsic immunomodulatory properties. MNMs possess enzyme-mimetic and antioxidant activities, allowing them to directly scavenge pathological levels of reactive oxygen species (ROS) within the OA joint. This activity, often mediated through surface ligand engineering that mimics natural enzymes, mitigates ROS-induced mitochondrial dysfunction in chondrocytes (36, 37). Evidence also indicates that MNMs can restore chondrocyte homeostasis by regulating pivotal ferroptosis pathways (38). Furthermore, surface-functionalized MNMs enable precise targeting of OA lesions and reprogram the immune microenvironment via modulation of specific immune cell subsets. More importantly, their programmable biointerface interactions can synergize with multiple treatment modalities—including chemotherapy, photothermal therapy, and gene therapy—to achieve precise intervention in the complex pathological microenvironment of OA (39). Collectively, these advancements signal a paradigm shift in OA management, moving from passive symptom control toward active reprogramming of the immune microenvironment, thereby offering a cross-disciplinary strategy to disrupt the self-perpetuating inflammation-oxidative stress cycle.

**Figure 1:**
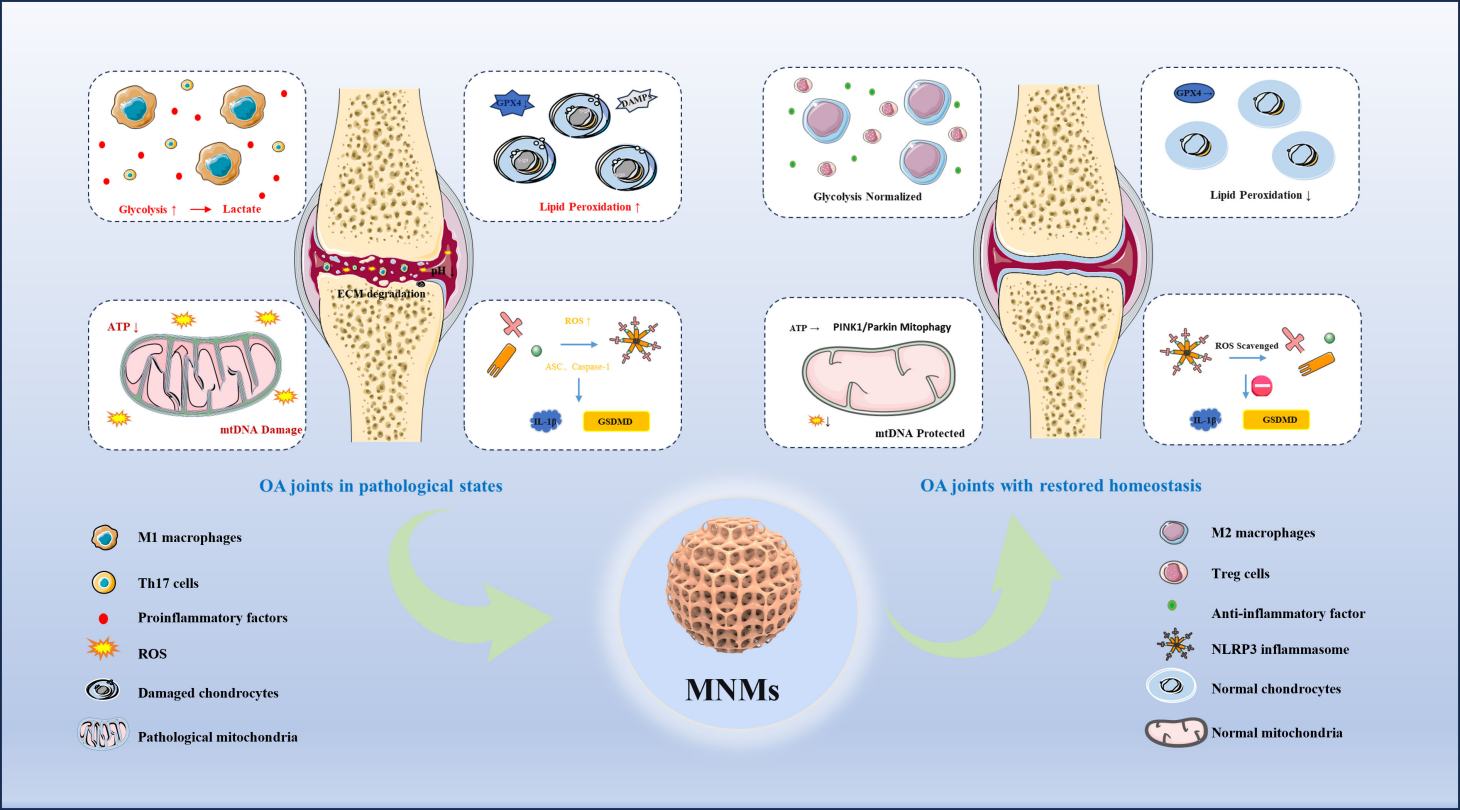
Schematic illustration of MNMs intervening in the osteoarthritic immunometabolic microenvironment through multi-modal metabolic reprogramming.

While several recent reviews have discussed the application of nanomaterials in osteoarthritis therapy, most have focused on a single material type (e.g., liposomes, polymeric nanoparticles) or emphasized traditional anti-inflammatory and cartilage repair mechanisms. They often lack a systematic synthesis of the unique immunometabolic regulatory capacities of MNMs and their latest advancements in multi-target, multi-modal synergistic treatment. Herein, to comprehensively assess recent advances in MNMs for OA immunotherapy and elucidate the advantages and limitations of such biomaterials in OA treatment, we summarize current applications of MNMs in OA immunotherapy (**Figure 1**). This review first outlines the immunopathology of OA and potential therapeutic targets, then details the immunomodulatory functions of MNMs. It subsequently examines the latest applications of MNMs in OA, discusses advanced design strategies and delivery systems in the third section, and finally critically assesses current research limitations and clinical challenges while providing perspectives on future directions. We believe this systematic review will serve as a valuable resource for research on MNM-based immunotherapy in OA.

## 2 Immunopathology and therapeutic targets in OA

### 2.1 M1/M2 macrophage polarization imbalance and synovial inflammation

Macrophages, as central regulators of the innate immune system, play a decisive role in OA synovial inflammation through their homeostatic polarization balance. Under physiological conditions, synovial macrophages (SMs) predominantly exhibit an M2 phenotype, maintaining joint homeostasis by secreting anti-inflammatory cytokines such as IL-10 and TGF-β (40). However, during OA progression, mechanical stress, damage-associated molecular patterns (DAMPs), and local hypoxic microenvironments drive macrophage polarization toward the M1 phenotype, establishing a pathogenic “inflammation-damage” positive feedback loop (41, 42). Recent single-cell RNA sequencing studies of human osteoarthritic synovium have identified distinct macrophage subpopulations with divergent functional roles. Notably, compared with healthy controls, a pro-inflammatory cluster expressing high levels of SPP1, IL1B, and CCL2 is significantly expanded in OA, whereas an anti-inflammatory cluster characterized by FOLR2 and TREM2 expression is relatively diminished. These pro-inflammatory cells highly express IL-1β, TNF-α, and inducible nitric oxide synthase (iNOS). These mediators activate NF-κB and STAT3 signaling pathways, leading to upregulation of matrix metalloproteinases (MMP-13 and ADAMTS-5), which directly degrade the extracellular matrix (ECM) of cartilage (43–47). Furthermore, M1-polarized macrophages amplify the inflammatory response by releasing chemokines such as CCL2 and CXCL10, which recruit monocytes to the synovium (48). Studies have confirmed that mechanical stress correction can significantly reduce the proportion of M1-polarized macrophages in the synovium, decrease IL-1β levels, and improve knee function in patients (49, 50). Conversely, M2 macrophage populations are quantitatively reduced in OA synovial tissue compared to healthy controls, and their functional impairment is linked to downregulated TREM2 (Triggering Receptor Expressed on Myeloid cells 2) expression (51). TREM2 suppresses M1 polarization by inhibiting the TLR4/MyD88 pathway (52), while excessive ROS in OA synovial fluid oxidatively modifies STAT6 to suppress M2 markers (e.g., CD163), exacerbating immune imbalance (53). Recent studies propose that modulating macrophage polarization through physical or chemical approaches can effectively alleviate synovial inflammation (54). These advances highlight the considerable potential of targeting macrophage polarization as a core strategy for OA immunotherapy.

### 2.2 Th17/Treg cell imbalance and cytokine storm

An imbalance in the Th17/Treg cell ratio within the adaptive immune system represents another core mechanism underlying the dysregulation of the OA immune microenvironment (55). Th17 cells secrete pro-inflammatory cytokines such as IL-17 and IL-21, whereas Treg cells maintain immune tolerance through TGF-β and IL-10 secretion. Disruption of this balance triggers a “cytokine storm” in OA lesions, which disrupts immune homeostasis and exacerbates cartilage degradation (56, 57). Studies have revealed significantly elevated Th17 cell proportions in the peripheral blood and synovial fluid of OA patients. The IL-17 secreted by these cells activates NF-κB and MAPK pathways, further inducing chondrocytes to express MMP-3, MMP-13, and ADAMTS-4, thereby promoting ECM degradation (58, 59). Meanwhile, IL-17 synergizes with IL-1β and TNF-α released by M1 macrophages to form an inflammatory amplification network (60). Moreover, OA patients exhibit reduced Treg cell numbers and impaired function, primarily due to compromised TGF-β signaling and IL-6-mediated STAT3 hyperactivation (61). IL-6 suppresses Foxp3 expression to inhibit Treg differentiation while promoting naïve T cell differentiation into Th17 cells, establishing a vicious cycle (62). Studies demonstrate that vitamin D3 deficiency disrupts the Th1/Th17/Th2 balance, thereby influencing degenerative joint disease progression (63). Additionally, inhibition of MiR-206 restores Th17/Treg equilibrium (64). These findings offer promising therapeutic targets for OA.

### 2.3 NLRP3 inflammasome activation and cartilage degradation

The NLRP3 inflammasome, a critical innate immune sensor, plays a key role in OA cartilage degradation through its aberrant activation. Integrating DAMPs and ROS signals, NLRP3 triggers caspase-1-dependent maturation and release of IL-1β and IL-18, directly disrupting cartilage homeostasis (65). LEF1 (Lymphoid Enhancer Binding Factor 1), a key transcription factor in the Wnt/β-catenin pathway, promotes inflammasome assembly by binding to the NACHT domain of NLRP3 (65–67). Activated NLRP3 further recruits ASC and pro-caspase-1 to form complexes that catalyze the cleavage of pro-IL-1β into its active form (68, 69). Concurrently, activated NLRP3 triggers Gasdermin D-mediated pyroptosis, exacerbating synovial inflammation and directly driving cartilage degradation (70). Notably, excessive ROS in the OA joint cavity not only directly impairs mitochondrial function but also amplifies inflammatory signaling through NLRP3 inflammasome activation (71). Therefore, ROS scavenging can simultaneously inhibit the NLRP3 pathway and reduce IL-1β levels, thereby protecting chondrocytes. Studies indicate that miR-140-5p suppresses chondrocyte phagocytosis and alleviates OA cartilage damage by inhibiting CTSB/NLRP3 (72). Furthermore, inhibition of the mitochondrial ROS/Cl- efflux signaling pathway effectively suppresses NLRP3 inflammasome activation, mitigating osteoarthritis (73). These findings provide a theoretical foundation for immunotherapy targeting OA cartilage damage.

### 2.4 Oxidative stress microenvironment

Oxidative stress (OS) is well-established as a critical contributor to the pathogenesis of OA. Recent studies have identified excessive accumulation of ROS as a key factor driving mitochondrial dysfunction, ferroptosis, and immune microenvironment imbalance in chondrocytes (74–76). ROS, including superoxide anion (O_2_^-^·), hydrogen peroxide (H_2_O_2_), and hydroxyl radical (·OH), are reactive molecules generated during oxygen metabolism (77). Chondrocytes primarily rely on mitochondrial oxidative phosphorylation for energy production; however, in OA, mitochondrial dysfunction reduces electron transport chain efficiency and increases electron leakage, promoting ROS generation (78). Furthermore, inflammatory cytokines activate NADPH oxidase (NOX) family enzymes, catalyzing NADPH oxidation to produce substantial O_2_^-^· (79). Additionally, expression of antioxidant enzymes such as superoxide dismutase (SOD) and glutathione peroxidase 4 (GPX4) is reduced in OA cartilage, impairing ROS clearance capacity (80). Through these combined mechanisms, excessive ROS directly damages chondrocyte DNA, proteins, and lipids, while concurrently activating NF-κB and MAPK signaling pathways to further promote inflammatory cytokine release, establishing a vicious “oxidative stress-inflammation” cycle. Consequently, targeting ROS has emerged as a novel strategic approach for osteoarthritis management (81).

### 2.5 Mitochondrial dysfunction

Mitochondria serve as both the primary source and target of ROS, with their dysfunction playing a central role in chondrocyte death in OA. Excessive ROS in the microenvironment attacks the mitochondrial inner membrane, leading to opening of the mitochondrial permeability transition pore (mPTP) and a subsequent decrease in mitochondrial membrane potential. This reduces ATP synthesis and ultimately triggers apoptosis (82, 83). Furthermore, mitochondrial mtDNA, lacking histone protection, is highly vulnerable to ROS attack, resulting in dysfunction of electron transport chain (ETC) proteins (e.g., Complex I and III) and further increasing ROS production (84). Recent studies have elucidated the role of impaired mitophagy in OA progression. The PINK1/Parkin pathway-mediated mitophagy, a key mechanism for clearing damaged mitochondria, is impaired in OA, leading to accumulation of dysfunctional mitochondria and sustained ROS release (85–87). These changes not only exacerbate the energy crisis in chondrocytes but also promote cartilage degradation by releasing cytochrome c and apoptosis-inducing factor (AIF), which activate caspase-dependent apoptotic pathways (88). Targeting the regulation of mitophagy has recently emerged as a promising therapeutic strategy for OA (89).

### 2.6 Chondrocyte ferroptosis

Ferroptosis is a novel form of regulated cell death characterized by intracellular iron overload and lethal accumulation of lipid peroxides (90). Accumulating evidence indicates that ROS and dysregulated iron metabolism synergistically promote ferroptosis in OA (91). In OA models, transferrin receptor 1 (TFR1) is upregulated, facilitating increased iron (Fe^2+^) influx (92). Additionally, enhanced ferritin degradation releases free iron, which generates excessive ·OH via the Fenton reaction, further aggravating lipid peroxidation (93). Glutathione peroxidase 4 (GPX4), a key antioxidant enzyme, relies on glutathione (GSH) to reduce lipid peroxides such as 4-HNE (94). In OA, however, ROS suppresses GPX4 expression and activity through methylation modifications, impairing its capacity to eliminate lipid ROS (95, 96). Ferroptosis not only directly causes chondrocyte death but also exacerbates synovial inflammation and immune dysregulation by releasing damage-associated molecular patterns that activate innate immunity (97). 4-octyl itaconate (4-OI) has been shown to protect chondrocytes from IL-1β-induced oxidative stress and ferroptosis by inhibiting GPX4 methylation (98). In recent years, targeting the ROS-ferroptosis axis has emerged as a promising strategy for the precise treatment of OA (99).

**Figure 2:**
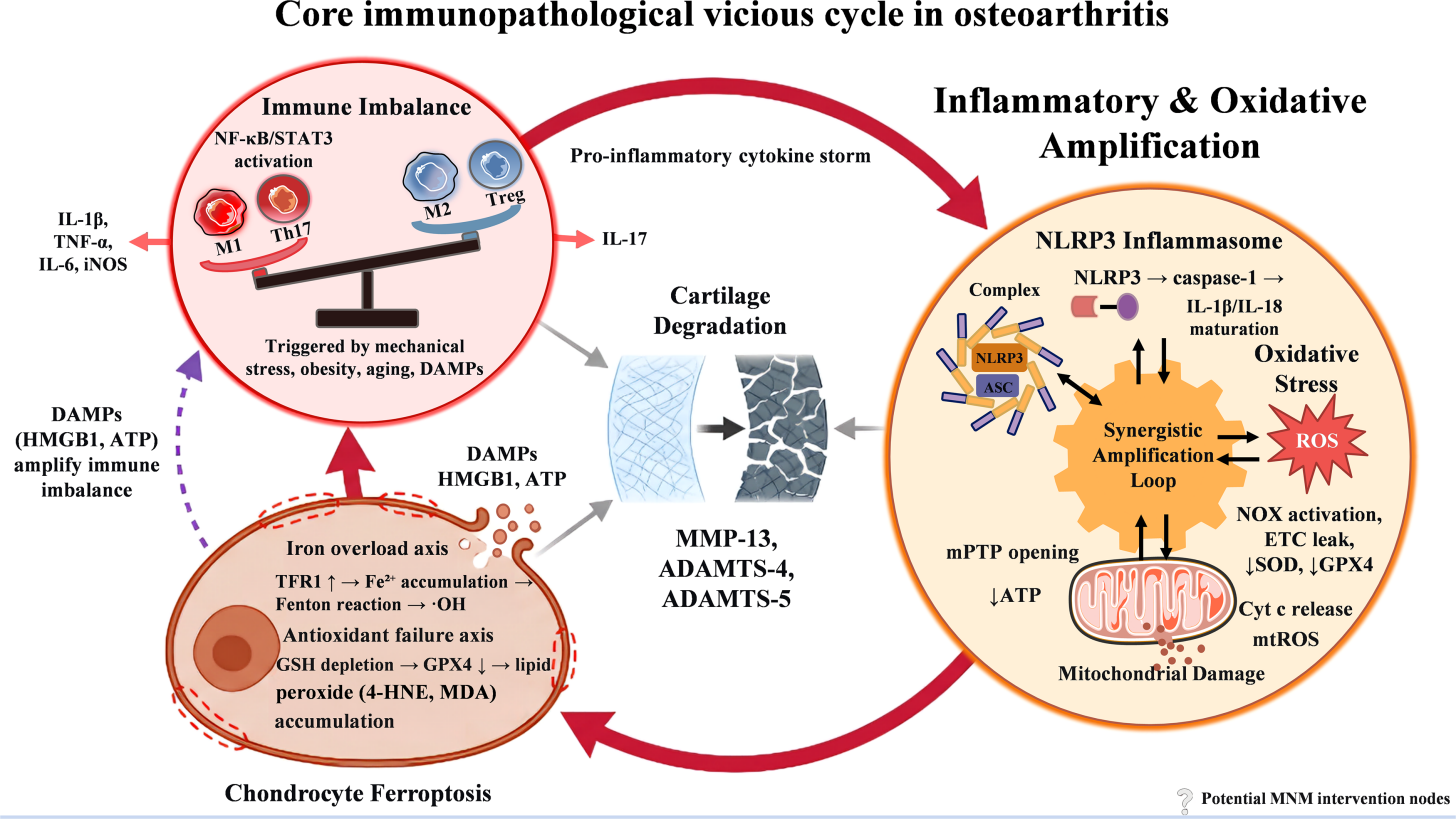
Core molecular targets within the OA immunopathological vicious cycle: a vicious cycle composed of immune cell imbalance, inflammasome activation, oxidative stress, and ferroptosis.

Collectively, these interconnected pathological mechanisms, which span macrophage polarization imbalance, adaptive immune dysregulation, NLRP3 inflammasome hyperactivation, oxidative stress, mitochondrial dysfunction, and chondrocyte ferroptosis, form a self-amplifying immunometabolic circuit that drives OA progression, as schematically summarized in **Figure 2**. The cascade is typically initiated by innate immune activation in response to mechanical microtrauma and damage-associated molecular patterns, or DAMPs, released from stressed chondrocytes, triggering M1 macrophage polarization and NLRP3 inflammasome assembly. The resulting pro-inflammatory milieu fuels oxidative stress through NOX enzyme activation and mitochondrial damage, which in turn exacerbates both NLRP3 activation and M1 polarization. Concurrently, impaired mitophagy and GPX4 suppression sensitize chondrocytes to ferroptotic death, releasing additional DAMPs that perpetuate the cycle. This integrated view positions immune-metabolic dysregulation not merely as an epiphenomenon of cartilage wear, but as a central driver of OA pathology, and thus represents a rational target for therapeutic intervention using MNMs.

## 3 Immunomodulatory functions of MNMs

**Figure 3:**
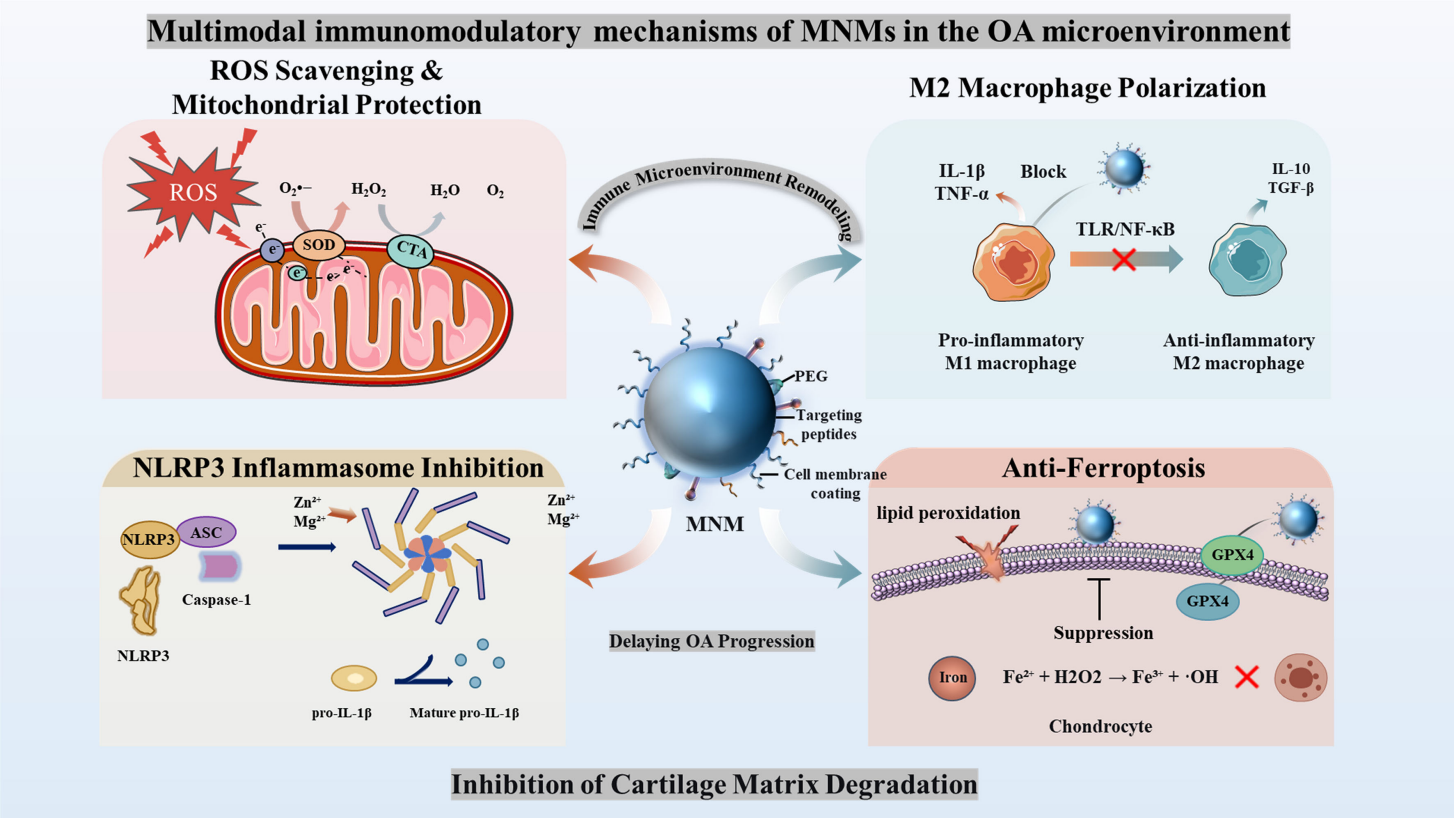
Multimodal immunomodulatory mechanisms of metal-based nanomaterials in the OA microenvironment:MNMs intervene in OA pathology through four principal axes: ROS scavenging and mitochondrial protection, macrophage M1-to-M2 repolarization, NLRP3 inflammasome inhibition, and attenuation of chondrocyte ferroptosis, collectively remodeling the immune–metabolic microenvironment.

Metal-based nanomaterials exert immunomodulatory effects in the OA joint through four interconnected mechanisms, as summarized in **Figure 3**. A detailed overview of representative MNMs, their mechanisms of action, and key preclinical outcomes is provided in **Table 1**. These include direct scavenging of reactive oxygen species with concomitant mitochondrial protection, reprogramming of synovial macrophages toward an anti-inflammatory M2 phenotype, specific blockade of NLRP3 inflammasome assembly and IL-1β maturation, and attenuation of chondrocyte ferroptosis via combined redox regulation and iron homeostasis control.

**Table 1:** Immunomodulatory functions and key research outcomes of representative MNMs in osteoarthritis therapy.

Category	Main function	Mechanism of action	Representative specific materials	Surface modification or Loaded cargo	Targeted cells/Signaling pathways	Validated models & Key therapeutic outcomes	Ref.
Antioxidant Materials	Scavenge ROS; Protect mitochondria; Inhibit cellular senescence	Mimic SOD/CAT enzymes; Reduce oxidative stress; Modulate PI3K/AKT & MAPK pathways	WY-PEG-CeO NPs (Spherical, ~100 nm)	PEGylation; Cartilage-targeting peptide (WYRGRL)	Chondrocytes; PI3K/AKT, MAPK pathways	Mouse OA model; Alleviated OA progression, reduced ECM degradation, no significant biotoxicity	(100–102)
	Scavenge H_2_O_2_; Establish dual antioxidant defense; Regulate osteoclast differentiation	Catalyze H_2_O_2_ decomposition; Upregulate SOD/GPx expression; Reduce osteoclast-associated ROS	M-PD Hydrogel (Mn-doped hydrogel, containing MnO_2_ NPs)	Incorporated into a hydrogel matrix for local delivery	Chondrocytes & Osteoclasts; ROS-mediated signaling	*In vitro* OA model; Selectively scavenged ROS, protected chondrocytes, inhibited osteoclast-associated ROS, remodeled the immune microenvironment	(81)
Macrophage-Regulating Materials	Promote M2 polarization; Suppress synovial inflammation	Suppress TLR/NF-κB pathway; Upregulate anti-inflammatory cytokines (IL-10, TGF-β); Leverage PTT for M1 depletion	Au-M2 NPs (Gold nanoparticles, spherical, ~50 nm)	Coated with M2 macrophage membrane	M1 Macrophages; Inflammatory cytokine (IL-1β, TNF-α) adsorption	IL-1β-induced ex vivo OA model (cartilage explants); Suppressed matrix degradation, reduced s-GAG loss and MMP13/NO production, outperformed M0/M1-coated particles	(138)
	Promote M2 polarization; Suppress inflammation; Coordinated cartilage-bone repair	Release Mg^2+^ to suppress NF-κB nuclear translocation; Activate AMPK-autophagy pathway; Inhibit osteoclasts while promoting osteogenesis	MENP (Mg^2+^-based biomimetic gene vector)	Co-loaded with siHIF-2α; M2 macrophage membrane coating	Macrophages, Chondrocytes; HIF-1α/HIF-2α, PI3K/AKT pathways	Rat OA model; Induced M1-to-M2 shift, significantly alleviated synovitis, promoted chondrocyte proliferation and coordinated cartilage-bone repair	(118, 119)
Inflammasome-Targeting Materials	Inhibit NLRP3 inflammasome activation; Block IL-1β/IL-18 maturation	Block NLRP3-ASC complex assembly; Suppress caspase-1 activation; Dual-blockade of ROS/NLRP3/IL-1β axis	MgO&SA@PLGA (pH-responsive microspheres encapsulating MgO NPs)	PLGA microspheres for pH-controlled release	Macrophages; NLRP3-ASC complex, ROS/NLRP3/IL-1β axis, AMPK pathway	Rat OA model; Reduced OARSI score by 45%, improved synovitis score by 60%, decreased osteophyte volume by 50%	(124)
	Inhibit NLRP3 inflammasome; Alleviate inflammation and oxidative stress	Mimic SOD to scavenge ROS; Bind HMGB1 to block TLR4; Downregulate inflammatory genes	CSP@AS-IV (Copper silicate NPs loaded with astragaloside IV, spherical)	Bioactive coating with astragaloside IV	Chondrocytes; Inflammatory cytokines (IL-6, TNF-α), antioxidant gene (Cat) upregulation	IL-1β-induced *in vitro* OA model; Significantly alleviated inflammation and oxidative stress, remodeled the inflammatory microenvironment	(130)
Metal-Based Theranostic Platforms	Combine multi-modal imaging with immunoprecise therapy; Real-time monitoring of tissue repair therapy	Enable high-resolution MRI imaging; Guide and monitor immunomodulatory therapy; Serve as dynamic feedback scaffold	Gd-HA NPs (Gadolinium-conjugated hyaluronic acid nanoparticles)	Hyaluronic acid for cartilage matrix targeting	Cartilage tissue; MRI signal-to-noise ratio enhancement	*In vivo* MRI model; Enhanced image contrast for fine structural visualization, enabling accurate diagnosis of early-stage synovitis	(133)
	Combine therapy with non-invasive monitoring of bone regeneration	Provide T2-weighted MRI contrast for scaffold tracking; Deliver VEGF to promote angiogenesis and osteogenesis	Fe_3_O_4_ Composite Hydrogel (Superparamagnetic scaffold)	Loaded with VEGF for concurrent tissue regeneration	Bone Marrow Mesenchymal Stem Cells (BMSCs); Osteogenic differentiation & neovascularization pathways	*In vivo* subchondral defect model; Facilitated robust bone repair, provided non-invasive MRI monitoring of regenerating bone replacing scaffold material	(134)

### 3.1 Antioxidant materials: ROS scavenging and mitochondrial protection

Excessive accumulation of ROS in the OA joint cavity is a key factor contributing to mitochondrial damage, ferroptosis, and inflammatory cascades in chondrocytes. Antioxidant MNMs can protect chondrocytes and restore immune homeostasis by either mimicking natural antioxidant enzymes (e.g., SOD, CAT) or directly scavenging ROS. Cerium dioxide (CeO_2_) exhibits dual enzyme-like activities—superoxide dismutase (SOD) and catalase (CAT)—due to its reversible Ce^3+^/Ce^4+^ redox transition, enabling stepwise scavenging of O_2_^-^· and H_2_O_2_ to mitigate oxidative damage. Its antioxidant capacity is orders of magnitude higher than that of conventional antioxidants (100). The Ce^3+^ sites catalyze the dismutation of O_2_^-^· to H_2_O_2_, while Ce^4+^ sites further decompose H_2_O_2_ into H_2_O, thereby inhibiting ROS-mediated activation of NF-κB and MAPK inflammatory pathways and demonstrating potent ROS scavenging capabilities (101). Furthermore, CeO_2_ can penetrate chondrocyte membranes and target mitochondria, where it modulates mitochondrial membrane potential and ATP synthesis to delay cellular senescence and apoptosis, thereby maintaining extracellular matrix (ECM) homeostasis. Zhuang et al. developed functionalized CeO_2_ nanoparticles (WY-PEG-CeO) encapsulated with polyethylene glycol (PEG) and conjugated with a cartilage-targeting peptide (WYRGRL), designed to address oxidative stress and chondrocyte senescence in OA (102). Their study revealed for the first time that WY-PEG-CeO exerts chondroprotective effects by synergistically combining antioxidant activity with suppression of the overactivated PI3K/AKT and MAPK signaling pathways. Animal experiments confirmed that this strategy effectively alleviates OA progression without significant biotoxicity. Similarly, Yang et al. constructed a bionic nanozyme platform named MHTCK based on cerium oxide, which integrates the redox activity of Ce^3+^/Ce^4+^ with the anti-inflammatory peptide KAFAK to innovatively target and modulate the oxidative and inflammatory microenvironment in OA (103). This work provides new evidence for the immunometabolic regulatory mechanisms of MNMs.

Manganese-based materials (e.g., MnO_2_) catalyze the decomposition of H_2_O_2_ into H_2_O and O_2_ via enzyme-mimetic activity while upregulating the expression of superoxide dismutase (SOD) and glutathione peroxidase (GPx), thereby establishing a dual antioxidant defense system (104). Jeon et al. developed a functionalized M-PD hydrogel based on manganese-doped nanomaterials (MnO_2_-PD), which utilizes the peroxidase-like activity of MnO_2_ to selectively scavenge excess ROS in the joint microenvironment. By reducing ROS levels associated with osteoclast differentiation and protecting chondrocytes from oxidative damage, this system innovatively achieves dual therapeutic goals: regulating oxidative stress and remodeling the immune microenvironment in OA treatment (81). Antioxidant MNMs are suitable for early intervention in OA subtypes with high oxidative stress; however, chronic use may cause toxicity due to metal ion accumulation, necessitating safety optimization through surface PEGylation or biodegradable designs. Moreover, as cell signaling requires moderate ROS levels, these materials must exhibit dynamic responsive release properties to achieve precise regulation (105).

### 3.2 Macrophage-regulating materials: M2 polarization induction and inflammation suppression

Imbalanced M1/M2 polarization of synovial macrophages is a core driver of chronic inflammation in OA, making macrophage repolarization a pivotal target for OA immunotherapy. MNMs can effectively reshape macrophage phenotypes and suppress synovitis through surface modifications or controlled ion release (106). Gold nanoparticles (Au NPs) suppress M1 macrophage activation and promote their transition to the anti-inflammatory M2 phenotype by modulating the Toll-like receptor (TLR)/NF-κB signaling pathway (107, 108). Studies demonstrate that Au NPs reduce levels of IL-6 and TNF-α in synovial fluid while upregulating IL-10 and TGF-β expression, thereby fostering a pro-repair microenvironment (109–111). Additionally, the surface plasmon resonance effect of Au NPs enhances the precision of photothermal therapy (PTT), enabling selective elimination of M1 macrophages through localized hyperthermia (112, 113).

Magnesium-based materials suppress the nuclear translocation of NF-κB via released Mg^2+^ ions, thereby blocking inflammatory cascades (114, 115). Mg^2+^ also activates the AMPK pathway to enhance autophagy and clear damaged mitochondria, reducing ROS production (116, 117). Zheng et al. developed a biomimetic gene delivery system named MENP, which simultaneously modulates HIF-1α and HIF-2α and optimizes macrophage polarization by targeted delivery of siHIF-2α and Mg^2+^ to inflamed OA joints. Their results indicate that Mg^2+^ not only promotes chondrocyte proliferation and differentiation but also induces a shift from pro-inflammatory M1 to anti-inflammatory M2 macrophages, significantly alleviating synovitis (118). In another study, Zheng’s team constructed a therapeutic system based on magnesium oxide nanoparticles (MgO NPs) that modulates macrophage polarization and multiple cellular behaviors to achieve coordinated cartilage-bone repair. The study revealed that Mg^2+^ suppresses pro-inflammatory factor expression via the PI3K/AKT pathway, mitigating inflammatory damage to cartilage. Notably, Mg^2+^ was shown to inhibit osteoclast formation and function—reducing bone resorption—while enhancing osteoblast activity to maintain bone metabolic balance. This work deepens the understanding of Mg^2+^ mechanisms in OA and provides a theoretical and technical foundation for novel treatment strategies (119). Therefore, macrophage-regulating materials are particularly suitable for mid-to-late-stage OA cases with significant synovitis. However, their efficacy may be influenced by particle size and surface charge, which affect macrophage phagocytosis, necessitating improved targeting through size optimization and surface modification. Additionally, long-term immunomodulation may cause immunosuppression, requiring combination with localized delivery systems to achieve spatiotemporally controlled release.

### 3.3 Inflammasome-targeting materials: NLRP3 pathway blockade and IL-1β inhibition

Overactivation of the NLRP3 inflammasome is a key driver of cartilage degradation in OA. Zinc oxide (ZnO) and copper-based (Cu) MNMs can specifically inhibit NLRP3 assembly and IL-1β maturation through ion release or surface interactions (120, 121). Studies demonstrate that Zn^2+^ blocks the assembly of the NLRP3-ASC complex and inhibits caspase-1 activation, thereby reducing the maturation and release of IL-1β and IL-18 (122). Additionally, ZnO NPs enhance cellular antioxidant capacity by modulating the Nrf2 signaling pathway, resulting in a dual-pathway inhibitory effect (123). Shu et al. developed a pH-responsive microsphere system (MgO&SA@PLGA) based on magnesium oxide nanoparticles for OA immunotherapy. Released Mg^2+^ ions target and suppress NLRP3 inflammasome activation, interrupting inflammatory cascades. Concurrently, intracellular Mg^2+^ modulates the ROS/NLRP3/IL-1β signaling axis and activates the AMPK pathway, achieving a “dual-blockade” effect on inflammasome activity. In a rat OA model, intra-articular injection of MgO&SA@PLGA reduced the OARSI score by 45%, improved the synovitis score by 60%, and decreased osteophyte volume by 50%, demonstrating significant therapeutic potential (124). Recently, He et al. constructed a hybrid nanodrug (Zn-Met/p65 siRNA) via zinc ion-coordinated self-assembly, offering an innovative “metal carrier-gene silencing-metabolic intervention” strategy for bidirectional regulation of inflammation and autophagy in OA treatment using MNMs (125).

Copper-based materials (e.g., Cu_2_O) scavenge ROS by mimicking superoxide dismutase (SOD) activity, thereby indirectly suppressing NLRP3 activation (126). Cu^2+^ also binds to the HMGB1 protein, blocking its interaction with the TLR4 receptor and inhibiting NF-κB pathway initiation (127, 128). Furthermore, Cu_2_O nanoparticles mitigate fibroblast-driven inflammatory responses by modulating fibroblast phenotypes (129). For instance, Yang et al. developed a novel nanodrug (CSP@AS-IV) based on copper silicate nanoparticles (CSP) loaded with astragaloside IV (AS-IV) for OA treatment (130). CSP@AS-IV significantly alleviated interleukin-1β (IL-1β)-induced inflammation and oxidative stress in chondrocytes, while remodeling the inflammatory microenvironment of articular cartilage through downregulation of inflammatory factors (e.g., IL-6, TNF-α) and upregulation of antioxidant genes (e.g., Cat). Similarly, Yu et al. engineered a multifunctional nanozyme based on a copper-based metal-organic framework (Cu MOF), which exhibits robust SOD-like, catalase (CAT)-like, and hydroxyl radical (·OH) scavenging activities (131). Leveraging the synergistic antioxidant properties of copper active centers and their ability to modulate macrophage polarization, this system enables effective immunotherapy for OA. Thus, inflammasome-targeting materials hold promise for targeted therapy in OA subgroups with high NLRP3 expression. However, systemic inhibition of NLRP3 may compromise host defense mechanisms, necessitating the use of joint cavity-targeted delivery systems for precise and localized treatment.

### 3.4 Metal-based theranostic platforms: a closed-loop revolution in immunoprecise intervention

Building upon the diverse immunomodulatory capabilities of MNMs discussed above, ranging from ROS scavenging and macrophage reprogramming to inflammasome inhibition, a critical challenge emerges: how to translate these mechanistic benefits into precisely targeted and dynamically monitorable therapeutic outcomes in the heterogeneous OA microenvironment. Addressing this challenge requires the integration of real-time diagnostic imaging with on-demand therapeutic delivery, thereby establishing a closed-loop paradigm that bridges immunomodulation and precision intervention. Metal-based theranostic platforms have emerged to meet this need, forging a new paradigm for immunoprecise therapy in OA through a tripartite integration of multimodal imaging guidance, pathological microenvironment-responsive drug release, and dynamic feedback on immunotherapeutic efficacy. The distribution of immune cells within osteoarthritic joints is highly heterogeneous (132), rendering systemic drug delivery inadequate for precise intervention. MNMs have established a new paradigm for immunoprecise therapy in OA through a tripartite integration of multimodal imaging guidance, pathological microenvironment-responsive drug release, and dynamic feedback on immunotherapeutic efficacy. Gadolinium (Gd^3+^), as a contrast agent, significantly enhances the signal-to-noise ratio in MRI. Specifically, gadolinium conjugated with hyaluronic acid (Gd-HA NPs) provides higher image contrast within a shorter time frame. Owing to their nanoscale size and high affinity for the cartilage matrix, Gd-HA NPs penetrate the cartilage surface and reach deep regions, enabling detailed visualization of structural changes and facilitating accurate diagnosis of early-stage synovitis (133). A novel hydrogel scaffold incorporating Fe_3_O_4_ nanoparticles serves as a dynamically monitorable theranostic platform for regenerative OA treatment (134). This scaffold exhibits robust subchondral bone repair capacity, where the porous structure of the inorganic particles facilitates efficient delivery of loaded vascular endothelial growth factor (VEGF). The incorporated Fe_3_O_4_ nanoparticles promote osteogenic differentiation of bone marrow mesenchymal stem cells and accelerate neovascularization. Moreover, magnetic resonance imaging revealed that Fe_3_O_4_ nanoparticles are gradually replaced by newly formed bone during subchondral defect repair, allowing non-invasive and radiation-free monitoring of bone regeneration in OA therapy.

## 4 Advanced design strategies and delivery systems

**Figure 4:**
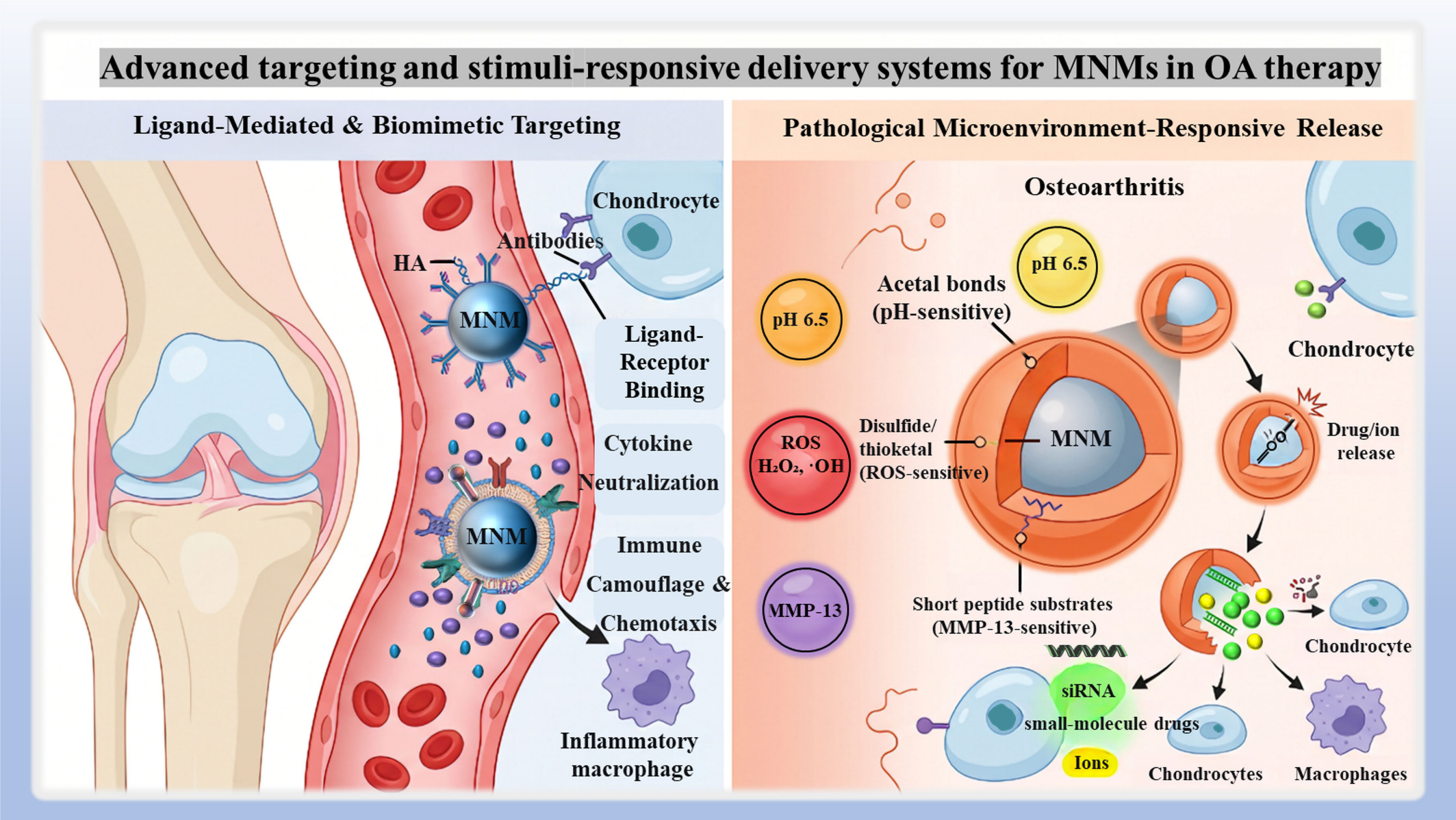
Advanced targeting and stimuli-responsive delivery strategies for metal-based nanomaterials in OA therapy:precision delivery is achieved by combining ligand-mediated/biomimetic active targeting with pathological microenvironment-responsive drug release, enabling lesion-specific accumulation and on-demand therapeutic cargo liberation.

Conventional OA immunotherapy is limited by poor targeting, low bioavailability, and significant off-target side effects. As illustrated in **Figure 4**, MNMs overcome these limitations through two complementary design strategies, namely ligand-mediated/biomimetic active targeting and microenvironment-responsive drug release, which together enable lesion-specific accumulation and spatiotemporally controlled therapeutic action. This section systematically elaborates these advanced delivery concepts and their recent applications in OA treatment.

### 4.1 Targeted modification strategy

#### 4.1.1 Ligand-mediated targeting and biomimetic surface engineering

Surface functionalization serves as a core strategy to enhance the targeting efficiency of MNMs for drug delivery. By modifying specific ligands or biomimetic membrane structures, it enables active accumulation of nanomaterials in inflamed joints. CD44, a hyaluronic acid (HA) receptor highly expressed on synovial cells and chondrocytes, is significantly upregulated in OA inflammatory regions (135). Conjugating HA to the surface of MNMs markedly enhances their intra-articular accumulation. For instance, researchers developed an empty self-assembled hyaluronic acid nanoparticle (HA-NP) for OA treatment (135). These nanoparticles specifically target the CD44 receptor, which is highly expressed in OA cartilage, and demonstrate excellent enzyme resistance and joint retention. Studies confirmed that HA-NP effectively blocks CD44-mediated activation of the NF-κB signaling pathway, significantly suppresses the catabolic activity of chondrocytes, and exhibits clear cartilage-protective effects in mouse OA models. This work not only provides a novel targeted therapeutic strategy but also elucidates the central role of the CD44–NF-κB axis in OA cartilage destruction. Furthermore, the hydrophilicity of the HA coating reduces non-specific adsorption of nanoparticles and prolongs their circulation time (136).

Recent studies have utilized macrophage membrane coating technology to confer inflammatory chemotactic capabilities to MNMs by preserving membrane surface chemokine receptors (e.g., CCR2, CX3CR1) (137). For example, researchers employed macrophage polarization techniques to fabricate gold nanoparticles (Au NPs) coated with membranes from macrophages of different polarization states (M0, M1, M2) for OA treatment (138). Among these, anti-inflammatory M2 macrophage membrane-coated nanoparticles (Au-M2 NPs) demonstrated exceptional inflammatory chemotactic ability and broad-spectrum pro-inflammatory cytokine adsorption (a “sponge” effect). In both *in vitro* and ex vivo OA models, Au-M2 NPs effectively cleared multiple pro-inflammatory cytokines, significantly alleviated inflammatory responses and cartilage matrix degradation, and performed markedly better than M0 or M1 membrane-coated particles. This study validates macrophage polarization as an effective strategy to enhance the immunotherapeutic efficacy of membrane-coated nanoparticles for OA, offering a novel approach for developing broad-spectrum anti-inflammatory nanotherapies targeting complex inflammatory environments.

Beyond ligand-mediated active targeting, the size and surface charge of MNMs are critical parameters governing their intra-articular fate. Cartilage ECM pore size restricts passive penetration to nanoparticles of approximately 60 nm or below in the superficial zone (139); particles exceeding this threshold are largely confined to the cartilage surface. Conversely, larger particles (e.g., 500–900 nm) show prolonged retention due to resistance to lymphatic drainage (140). Surface charge further modulates this behavior: cationic nanoparticles exploit electrostatic interactions with the negatively charged GAGs in cartilage, achieving enhanced penetration and retention, as demonstrated by avidin and PLL-PCL-modified TGFα nanoparticles (141). However, OA-associated proteoglycan depletion may reduce this electrostatic advantage by up to 3-fold (142, 143), and excess positive charge raises cytotoxicity concerns (144). Neutral or PEGylated surfaces improve colloidal stability and minimize off-target interactions. These size and charge profiles should therefore be tailored to the therapeutic objective—deep cartilage penetration versus prolonged synovial residence—and to the disease stage, given that ECM composition and charge density evolve during OA progression.

#### 4.1.2 Microenvironment-responsive release for precision-controlled drug delivery

The pathological microenvironment of OA possesses unique physicochemical characteristics (e.g., acidic pH, elevated ROS, MMP-13 overexpression), which can be leveraged by MNMs designed with responsive functional groups for on-demand drug release (145). The pH of inflamed synovial fluid (~6.5) is significantly lower than that of normal tissue (~7.4), while ROS levels can be elevated by more than 10-fold. For instance, researchers developed a pH-responsive metal-organic framework (MIL-101-NH_2_) for targeted OA therapy. This carrier intelligently disintegrates in the acidic joint microenvironment, synchronously releasing the anti-inflammatory drug curcumin (CCM) and HIF-2α-targeting siRNA, achieving synergistic “anti-inflammatory and gene silencing” therapy (146). Additionally, a ROS-responsive nanofibrous membrane (PPGF) was designed for OA treatment. This material utilizes PEGDA-EDT disulfide bonds as a ROS-cleavable switch, loaded with the antioxidant/anti-inflammatory agent fucoxanthin (Fx) and an rGO carrier. In the high-ROS OA microenvironment, PPGF specifically triggers Fx release, enabling ultra-long-term sustained release (>66 days) and precise synergistic antioxidant and anti-inflammatory therapy (147–149). Similarly, matrix metalloproteinases (MMPs), particularly the key target MMP-13, are overexpressed during OA cartilage degradation. For example, an MMP13 enzyme-responsive theranostic micelle (ERMs@siM13) was developed for post-traumatic osteoarthritis (PTOA) treatment. This material undergoes specific enzymatic cleavage in cartilage tissues overexpressing MMP13, leading to shedding of its PEG shell and exposure of cRGD targeting ligands. This promotes the highly efficient uptake of encapsulated MMP13 siRNA (siM13) by diseased chondrocytes, providing a paradigm for MMP-targeted smart theranostic materials (150).

### 4.2 Synergistic therapy systems

#### 4.2.1 Combination of photothermal therapy and immunomodulation

Photothermal therapy (PTT) induces localized hyperthermia to eliminate inflammatory cells, while the photothermal conversion properties of MNMs can synergize with immunomodulation to achieve a dual “hyperthermia-immunity” therapeutic effect (151, 152). For instance, researchers developed TRPV1 antibody-modified gold nanorods (Cit-AuNRs@Anti-TRPV1) for synergistic photothermal OA treatment (38). This construct targets the TRPV1 channel on chondrocytes via the antibody and, under NIR irradiation, converts light into localized heat to precisely activate TRPV1, thereby inhibiting chondrocyte ferroptosis. Furthermore, a magnesium ion-doped dual-biomimetic photothermal nanozyme (MPMP) was developed for synergistic OA therapy (153). Based on an MoS_2_ substrate and coated with polysulfobetaine, this material achieves enhanced lubrication (friction coefficient: 0.028) and mimics antioxidant enzymes to efficiently scavenge ROS/RNS (>90%). Under NIR irradiation, it triggers localized thermal effects and releases Mg^2+^, which suppresses the NF-κB/IL-17 pathway and activates the MAPK pathway to promote cartilage regeneration. Concurrently, it upregulates HSP70 and mimics hyaluronic acid synthase activity, significantly increasing HA synthesis (2-fold intracellularly, 3.12-fold extracellularly). In a mouse OA model, this system demonstrated an 83.41% reduction in osteophyte formation and an 88.57% decrease in OARSI score, confirming the therapeutic potential of metallic nanomaterials to remodel joint homeostasis through photothermal-immunological synergy.

Despite promising preclinical results, the clinical translation of PTT-based MNM platforms for OA faces a fundamental physical constraint: the limited penetration depth of near-infrared light in human tissues. Although NIR wavelengths (typically 650–950 nm) achieve deeper tissue penetration than visible light, the effective therapeutic depth in highly scattering biological tissues is generally limited to approximately 1–2 cm, which is insufficient to reach the entire joint space of large human articulations such as the knee or hip. Additionally, the presence of synovial fluid, dense connective tissue, and overlying muscle and adipose layers further attenuates light transmission. For these reasons, NIR-mediated PTT in clinical OA management is likely limited to adjuvant ablation of superficial inflamed synovium and may not be suitable as a standalone therapy for deep cartilage lesions. Alternative energy delivery modalities, such as ultrasound-triggered or alternating magnetic field-responsive hyperthermia, may offer more clinically viable approaches for deep-tissue targeting.

#### 4.2.2 Anti-inflammatory and immunomodulatory synergistic therapy: targeted delivery and chondrocyte pathological state reprogramming

Traditional anti-inflammatory drugs (e.g., IL-1β inhibitors, JAK inhibitors) can alleviate OA inflammation but suffer from off-target toxicity when administered systemically and rarely reverse the imbalanced immune microenvironment. MNMs enable a synergistic “anti-inflammatory and immune remodeling” effect through the precise co-delivery of anti-inflammatory agents and immunomodulatory factors. Huang et al. developed a folic acid-modified zirconium-based MOF nanocarrier (Bai@FA-UIO-66-NH_2_) that targets M1 macrophages to simultaneously scavenge ROS and regulate M1/M2 polarization, significantly mitigating synovial inflammation and cartilage degradation in osteoarthritis (154). In contrast to macrophage-targeted immunomodulation, an alternative cutting-edge strategy aims to directly reverse the pathological state of diseased chondrocytes, thereby indirectly reshaping the local immune microenvironment. While chondrocytes are structural cells rather than professional immune cells, they actively participate in OA-associated immune dysregulation by secreting pro-inflammatory cytokines (e.g., IL-1β, IL-6) and matrix-degrading enzymes that perpetuate inflammation and cartilage breakdown. Therefore, therapeutically restoring chondrocyte homeostasis represents an indirect but critical axis of immunomodulation in OA. One study constructed a novel metal-organic framework (MOF) nanoplatform (miR/IrO_2_@ZIF-8) that synergizes reactive oxygen species (ROS) scavenging with gene silencing. The ZIF-8 carrier was co-loaded with an antagonist antagomiR-181a (an antisense oligonucleotide, ASO). Through efficient delivery and lysosomal escape, the ASO precisely silences miR-181a—a key intracellular switch promoting inflammation and catabolism in chondrocytes. This intervention in the cellular gene expression program successfully downregulated the expression of inflammatory factors such as interleukins (IL-1β, IL-6) and cyclooxygenase-2 (COX-2), as well as cartilage-degrading enzymes including matrix metalloproteinase 13 (MMP13) and a disintegrin and metalloproteinase with thrombospondin motifs 5 (ADAMTS-5). Consequently, this approach reprograms chondrocytes away from a destructive, catabolic state toward homeostasis. This dual-track strategy—combining oxidative stress clearance with intrinsic cellular gene program regulation—enables synergistic osteoarthritis therapy and provides a robust paradigm for developing precision nanotherapies capable of directly restoring the function of diseased cells (155).

## 5 Challenges in clinical translation and future perspectives

**Figure 5:**
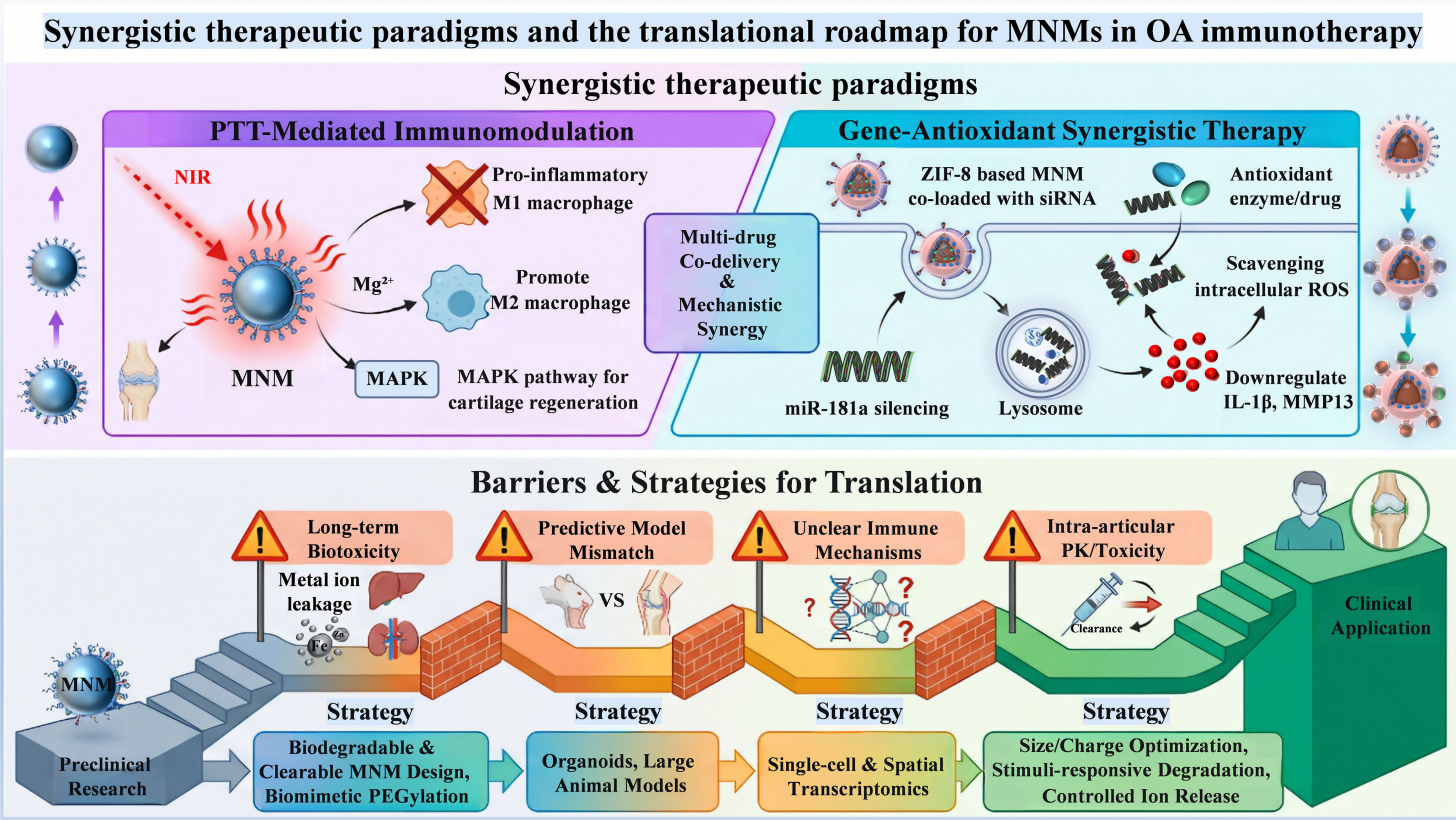
Synergistic therapeutic paradigms and translational roadmap for metal-based nanomaterial-mediated OA immunotherapy:current preclinical innovations integrate photothermal–immunomodulatory synergy and gene–antioxidant combination therapy; successful clinical translation requires overcoming barriers in biosafety, predictive models, mechanistic understanding, and intra-articular pharmacokinetics.

As summarized in **Figure 5**, synergistic platforms such as photothermal–immunomodulatory therapy and gene–antioxidant co-delivery have demonstrated potent disease-modifying effects in preclinical models. Despite this promise, clinical translation of MNM-based OA immunotherapy must overcome critical barriers—long-term biosafety, predictive model fidelity, mechanistic understanding, and intra-articular pharmacokinetics. This section examines these hurdles in depth and outlines potential strategies to address them.

### 5.1 Current barriers and mitigation strategies

Biosafety remains a central challenge in the clinical translation of MNMs, primarily due to toxicity from metal ion release, long-term bioaccumulation, and associated risks. For instance, silver nanoparticles (AgNPs) release silver ions, which exhibit notable neurotoxicity and may cause neural cell damage and dysfunction. AuNPs tend to accumulate in the liver and kidneys, potentially impairing their functions. Although iron-based nanoparticles (FeNPs) demonstrate relatively high biocompatibility, repeated or prolonged exposure may lead to iron accumulation in tissues, inducing oxidative stress and cytotoxicity (156). These long-term toxicity concerns significantly restrict the clinical applicability of MNMs. To address these challenges, several strategies have been proposed to improve biosafety. Surface modification is an effective approach—introducing biomimetic coatings (e.g., polyethylene glycol, chitosan) can enhance biocompatibility, reduce ion leakage, and mitigate systemic toxicity. Furthermore, surface functionalization improves the targeting ability of MNMs, enabling more precise accumulation at disease sites and reducing off-target effects on healthy tissues (157). Dosage and size optimization represent another critical strategy. By carefully controlling the administered dose and particle dimensions, a balance between therapeutic efficacy and safety can be achieved. Studies indicate that low doses and smaller-sized MNMs can maintain therapeutic effects while significantly reducing adverse reactions (158). Moreover, the design of biodegradable MNMs presents a promising direction. Such materials can be metabolized and cleared from the body after fulfilling their therapeutic function, thereby minimizing risks associated with long-term accumulation (159).

Furthermore, the pharmacokinetic profile of MNMs within the unique enclosed joint microenvironment warrants deeper investigation ahead of clinical translation. Following intra-articular injection, the clearance of nanoparticles from the joint cavity is primarily governed by two parallel pathways: drainage through the synovial capillary network and elimination via the lymphatic system (160). Small particles and released metal ions tend to exit the joint preferentially through capillaries, whereas larger nanoparticles and their degradation products are predominantly cleared via lymphatic drainage, regardless of size (161, 162). Importantly, the rate of synovial lymph flow is markedly enhanced under the systemic inflammatory and autoimmune conditions characteristic of RA and advanced OA, which may paradoxically accelerate the clearance of therapeutic MNMs from inflamed joints and diminish their therapeutic efficacy (163). The prolonged retention time of MNMs in the joint, although advantageous for sustained immunomodulation, also raises concerns regarding chronic metal ion release and localized metallosis. Non-biodegradable or slowly degradable metallic nanoparticles may continuously leach metal ions into the synovial fluid and adjacent cartilage tissue, potentially triggering metal ion-induced hypersensitivity reactions or periarticular tissue damage over extended periods. This risk is particularly pertinent for metal elements known to accumulate in tissues, such as cobalt and chromium. The degradation kinetics of MNMs in the pathologically altered joint microenvironment, where elevated levels of inflammatory cytokines, MMPs, and ROS coexist, remain largely unexplored. A comprehensive understanding of the degradation mechanisms, ion release profiles, and ultimate clearance pathways of MNMs within the arthritic joint is therefore essential to predict and mitigate long-term local toxicity risks and to rationally design next-generation nanomaterials with tailored biodegradation rates that match the therapeutic window required for OA immunotherapy.

Currently, preclinical research on MNMs for OA immunotherapy still heavily relies on small animal models, such as mice and rats. However, significant differences in joint structure, immune microenvironment, and disease progression between these models and human OA limit the reliability of translating such findings into clinical applications (164). Due to fundamental differences in immune response mechanisms and cartilage physiology between rodents and humans, therapeutic effects observed in small animal models are often difficult to replicate in patients (165). Notably, the most widely used preclinical models rely on surgical induction approaches, such as destabilization of the medial meniscus (DMM) or anterior cruciate ligament transection (ACLT), which primarily recapitulate post-traumatic OA. These models differ fundamentally from the spontaneous, age-related OA that predominates in humans, both in their temporal kinetics (acute onset over weeks versus gradual progression over decades) and in their underlying drivers (mechanical instability versus chronic low-grade inflammation and metabolic stress). In addition, human articular cartilage is substantially thicker (2–4 mm in the knee) than that of rodents (~0.15 mm), and the biomechanical loading patterns of bipedal humans differ markedly from quadrupedal rodents, further limiting the predictive value of these models for MNM pharmacokinetics and therapeutic efficacy. To enhance the clinical predictive value of research outcomes, more advanced model systems should be adopted. Organoids, as three-dimensional *in vitro* models, can closely mimic the complex structure and function of human tissues, providing a robust platform for investigating OA pathogenesis and drug screening (166, 167). For example, articular cartilage organoids enable more accurate evaluation of the effects of MNMs on cartilage repair, immunomodulation, and extracellular matrix homeostasis (168). On the other hand, large animal models (e.g., pigs, sheep) resemble humans more closely in terms of joint anatomy, biomechanical environment, and immune system, allowing for more faithful recapitulation of OA disease progression (169, 170). The use of large animal models not only permits comprehensive assessment of the therapeutic efficacy and biosafety of MNMs but also facilitates the investigation of their pharmacokinetic profiles and potential toxicity during long-term intervention, thereby providing more reliable data to support subsequent clinical studies.

Although MNMs demonstrate considerable potential for immunotherapy in OA, their molecular mechanisms and interactions with the immune system remain incompletely understood. Current research has primarily focused on the physicochemical properties and phenotypic therapeutic outcomes of MNMs, while systematic investigations into their immunomodulatory mechanisms are still limited. This knowledge gap significantly hinders the clinical translation of such materials. Single-cell RNA sequencing (scRNA-seq) offers a powerful tool to address this bottleneck. This technology enables high-resolution, high-throughput analysis of gene expression profiles at the single-cell level, allowing precise identification of dynamic changes in immune cell subsets and key signaling pathways in response to MNM treatment (171). For example, scRNA-seq can reveal specific immune cell types and functional state transitions modulated by MNMs during OA therapy, providing deep insights into their immunoregulatory mechanisms (172). Furthermore, spatial transcriptomics retains spatial information of cells while capturing transcriptomic data, facilitating *in situ* investigation of MNM distribution and their interactions with cells within the tissue microenvironment. This technique allows accurate assessment of MNM retention and distribution in articular cartilage and synovium, and elucidates spatial proximity and potential regulatory mechanisms between MNMs, chondrocytes, and immune cells. These insights provide critical evidence for understanding the targeting and immunomodulatory mechanisms of MNMs (173, 174).

Despite these challenges, the translational potential of MNMs in orthopedics and rheumatology is supported by existing clinical precedents for related nanomaterials. Iron oxide nanoparticles (IONPs), for instance, have been approved by the U.S. Food and Drug Administration (FDA) for clinical use as MRI contrast agents and, in certain formulations, for the treatment of iron deficiency anemia (175). More recently, a longitudinal clinical study demonstrated that daily oral supplementation with gold nanoparticles (AuNPs) at a micro-dosage of 0.34 mg elemental gold significantly improved the Knee injury and Osteoarthritis Outcome Score (KOOS) in a cohort of 51 arthritis patients over 20 weeks, with the majority of participants reporting reduced knee pain and improved joint function (176). Notably, this trial was the first to establish the safety and efficacy of low-dose AuNP supplementation for both rheumatoid arthritis and osteoarthritis patients, providing direct clinical evidence for the therapeutic potential of metal-based nanomaterials in arthritis management. Furthermore, intra-articular formulations such as FX006 (Zilretta), a PLGA-based extended-release microsphere encapsulating triamcinolone acetonide, have received FDA approval for knee OA treatment (177). Although FX006 is a polymeric microparticle rather than a metallic nanosystem, its successful clinical translation demonstrates the feasibility of designing particulate systems that achieve sustained drug release and prolonged joint retention, key objectives shared by MNM-based OA immunotherapies. These clinical precedents, spanning imaging, oral therapeutic, and intra-articular drug delivery applications, provide a realistic and promising translational pathway for next-generation MNMs designed to achieve multimodal immunomodulation in OA. Their regulatory and clinical successes also offer valuable benchmarks for addressing the safety, manufacturing, and efficacy evaluation challenges that currently hinder the clinical advancement of MNM-based OA therapies. Beyond these technical hurdles, a more fundamental consideration for clinical translation is the inherent heterogeneity of the disease itself.

OA is not a single disease entity but a heterogeneous syndrome encompassing distinct clinical phenotypes driven by divergent pathophysiological mechanisms. These phenotypes include: (i) an inflammatory phenotype, characterized by pronounced synovitis, joint effusion, and elevated inflammatory biomarkers, in which NLRP3 inflammasome activation and M1-like macrophage predominance are prominent; (ii) a metabolic phenotype, typically associated with obesity and metabolic syndrome, where systemic low-grade inflammation, adipokine dysregulation, and excessive mechanical loading converge; (iii) a biomechanical/post-traumatic phenotype, triggered by joint injury, instability, or malalignment, in which aberrant mechanotransduction and mitochondrial dysfunction predominate; and (iv) an age-related phenotype, marked by cellular senescence and impaired autophagy. Given this heterogeneity, MNM-based therapeutic strategies should be rationally tailored to the dominant pathological driver of each subtype. For example, NLRP3 inflammasome-targeting MNMs are likely to exhibit maximal efficacy in the inflammatory phenotype, whereas antioxidant and mitophagy-enhancing MNMs may be better suited for biomechanical or age-related subtypes. Anti-ferroptosis strategies, by contrast, may hold broader applicability, as oxidative stress represents a shared downstream pathway across phenotypes. Defining such therapeutic boundaries through molecular endotype-based patient stratification—ideally guided by synovial fluid biomarkers or advanced imaging features—will be critical for the successful clinical translation of MNM-based immunomodulatory therapies for OA.

## 6 Conclusions and outlook

MNMs demonstrate considerable advantages and broad application prospects in the immunotherapy of OA. Their core strengths are reflected in three key aspects: Multi-target regulatory capacity—simultaneously modulating inflammatory responses, oxidative stress, cell death, and other pathological processes, thereby breaking the “inflammation–oxidation” vicious cycle; High visualizability and potential for theranostics—components such as gadolinium- or iron oxide-based MNMs enabling real-time imaging and treatment monitoring, supporting prospects for precision medicine; Programmability and environmentally responsive behavior—through surface functionalization and microenvironment-triggered design, MNMs can achieve on-demand drug release or immunomodulation within specific pathological contexts, significantly improving targeting and safety.

However, achieving clinical translation will require deeper interdisciplinary collaboration. Close integration among materials science, immunology, and clinical medicine is essential to advance the field. Materials scientists must develop nanostructures with enhanced biocompatibility, biodegradability, and targeting specificity; immunologists should elucidate the molecular mechanisms underlying MNM–immune microenvironment interactions; and clinicians ought to contribute practical insights to develop more clinically relevant models and therapeutic strategies. Only through such collaborative efforts can the transition from basic research to clinical application be accelerated.

Looking forward, we propose the following research framework and translational pathway: First, establish a multi-level, cross-species research system integrating organoids, large animal models, and human samples to improve clinical predictive validity. Second, employ artificial intelligence and computational materials science to rationally design novel MNMs—for instance, using machine learning to predict immunomodulatory activity and biosafety (178). Finally, innovate clinical research paradigms by incorporating emerging technologies such as radiomics and liquid biopsy to construct robust efficacy evaluation and long-term follow-up systems for MNM-based therapies. Together, these approaches will form a closed translational loop encompassing “precision design – mechanistic validation – clinical evaluation.” Through sustained multidisciplinary collaboration and technological innovation, MNMs are poised to become a transformative force in future OA treatment.
